# Mollaret Meningitis Caused by Varicella-Zoster Virus: A Case Report

**DOI:** 10.7759/cureus.31834

**Published:** 2022-11-23

**Authors:** Haruna Akanuma, Shin-ichi Ueno, Shoko Tanaka, Nozomu Matsuda, Kazuaki Kanai

**Affiliations:** 1 Neurology, Fukushima Medical University School of Medicine, Fukushima, JPN; 2 Neurology, Juntendo University School of Medicine, Tokyo, JPN

**Keywords:** igg index, il-1β, familial mediterranean fever, varicella-zoster virus, mollaret's meningitis

## Abstract

Mollaret meningitis is a recurrent aseptic meningitis mostly caused by herpes simplex virus type 2. Other causes of the disease rarely exist, and its pathology is not well understood. Herein, we present a 57-year-old man who had been admitted to our hospital eight times with recurrent aseptic meningitis. Although the deoxyribonucleic acid (DNA) of varicella-zoster virus (VZV) was not detected in the cerebrospinal fluid (CSF), his genetic analysis, measurement of anti-VZV immunoglobulin-G (IgG) in the CSF, the VZV IgG index, IgG in the serum, and interleukin-1 beta in the CSF revealed that the Mollaret meningitis had been caused by the VZV. This case demonstrates that Mollaret meningitis can be caused by the VZV when specific factors are associated with decreased immune response. This case is valuable in elucidating the pathophysiology of Mollaret meningitis.

## Introduction

Mollaret meningitis is a recurrent aseptic meningitis (RAM), which was first reported by Mollaret in 1944 [1]. The disease is primarily caused by herpes simplex virus type 2 (HSV-2). However, other causes have been reported [2]. Mollaret meningitis has also been associated with familial Mediterranean fever (FMF) [2]. To the best of our knowledge, only one published report of Mollaret meningitis associated with varicella-zoster virus (VZV) reactivation is available [3].

The pathology of Mollaret meningitis is not well understood. However, in recent years, immune disorders have been reported to be associated with disease onset [4,5]. We report a case of Mollaret meningitis caused by VZV and associated with the decreased immune response against VZV.

## Case presentation

In November 2020, a 56-year-old man was admitted to our hospital with the eighth recurrence of aseptic meningitis. During this recurrence, neurological findings showed only meningeal signs. No obvious rash or genital herpes was observed. No markers of an inflammatory response, including serum amyloid A, were observed in the serum. The cerebrospinal fluid (CSF) contained 205 mononuclear cells/mm^3^ without malignant or Mollaret cells, 74 mg/dL protein, and 62 mg/dL glucose, and an oligoclonal band was seen. We started treatment with acyclovir, and the fever went down and the headache improved. In the follow-up CSF evaluation, the pleocytosis had improved.

**Figure 1:**
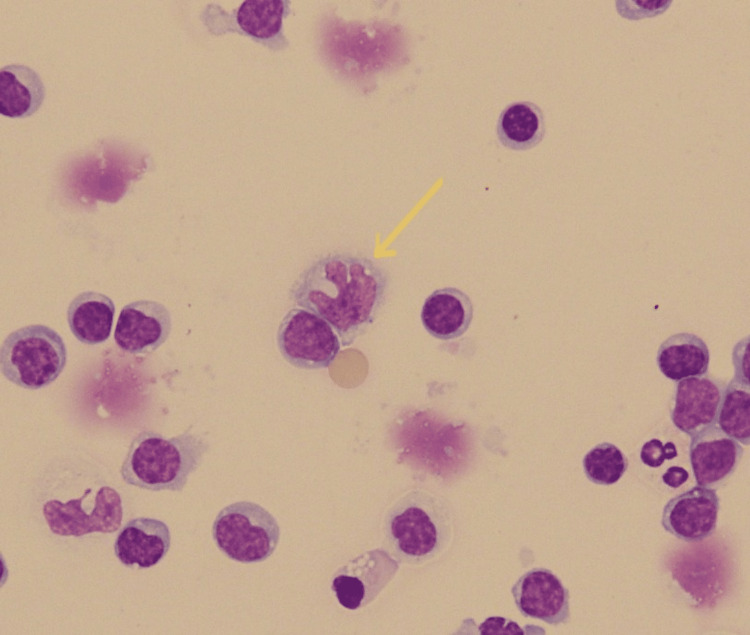
Giemsa staining showing Mollaret's cell (indicated by the yellow arrow) Mollaret's cell was a large monocyte-like cell with irregular “cloverleaf”-shaped nuclei.

The patient suffered the first episode of aseptic meningitis at the age of 39 years. Recurrences have occurred at intervals of one to four years. No bacterial or viral deoxyribonucleic acid (DNA) has been detected in the CSF to date. The patient had always visited the hospital with headache and fever for several days, and only signs of meningeal irritation were observed as physical findings. The CSF contained about 200 mononuclear cells/mm^3^ cell count at the extreme stage of recurrences, and improvements in CSF findings can be obtained along with improvements in clinical symptoms. Mollaret's cell was detected in the seventh recurrence (Figure 1). Cranial magnetic resonance imaging was normal. Antinuclear antibodies and antibodies for anti-Sjögren’s-syndrome-related antigens A and B, anti-U1 ribonucleoprotein, myeloperoxidase anti-neutrophil cytoplasmic, serine proteinase 3-antineutrophil cytoplasmic, anti-aquaporin 4, and nonspecific antibodies (such as rheumatoid factor) were not detected. Human leukocyte antigen (HLA) haplotype analysis revealed the presence of HLA A24 and B7/B52, and we had no specific physical findings. We, therefore, considered that the probability of Behçet’s disease was low. Genetic analysis revealed the heterozygous genotype with the E148Q mutation in the familial Mediterranean fever (MEFV) gene that causes FMF [6]; however, colchicine, 1 mg per day, did not prevent the recurrence. Because of the lack of physical findings characteristic of FMF other than a headache and the ineffectiveness of colchicine, we suspected that this patient did not have FMF. The dose of colchicine could not be increased to 2 mg/day because of gastrointestinal symptoms.

During the seventh and eighth recurrences, anti-VZV immunoglobulin-G (IgG) (enzyme immunoassay) in the CSF increased by 0.7 and 0.9, respectively (normal range: <0.2), while the VZV IgG index (CSF virus-specific IgG titer/serum titer) increased by 3.56 and 0.9, respectively (normal range: <1.5-2.0) [7]. As the patient’s symptoms and clinical course fulfilled the diagnostic criteria for Mollaret meningitis reported by Bruyn et al. (Table 1) [8] and Galdi (Table 2) [9], we diagnosed the condition as Mollaret meningitis. In addition, anti-HSV-2 IgG was not detected in the CSF, and findings suggestive of VZV infection were observed; therefore, Mollaret meningitis caused by VZV was broadly suspected.

**Table 1:** Diagnostic criteria of Mollaret meningitis by Bruyn et al. in 1962 CSF: Cerebrospinal fluid. Source: Ref. [8].

Diagnostic criteria
(1) Recurrent attacks of fever are associated with signs and symptoms of meningeal irritation.
(2) The attacks are separated by symptom-free intervals lasting for weeks or months.
(3) During the attacks, there is CSF pleocytosis of a mixed type, including endothelial cells, leukocytes, and lymphocytes.
(4) The disease is followed by remission without residual signs.
(5) No causative organism can be detected.

**Table 2:** Diagnostic criteria of Mollaret meningitis by Galdi in 1979 CSF: Cerebrospinal fluid. Source: Ref. [9].

Diagnostic criteria
(1) Fever is not necessarily present.
(2) Approximately 50% of the patients will have transitory neurologic symptoms or signs in addition to those of meningeal irritation.
(3) The symptom-free intervals may vary from days to years.
(4) There may be an increased γ-globulin fraction in the CSF.

**Figure 2:**
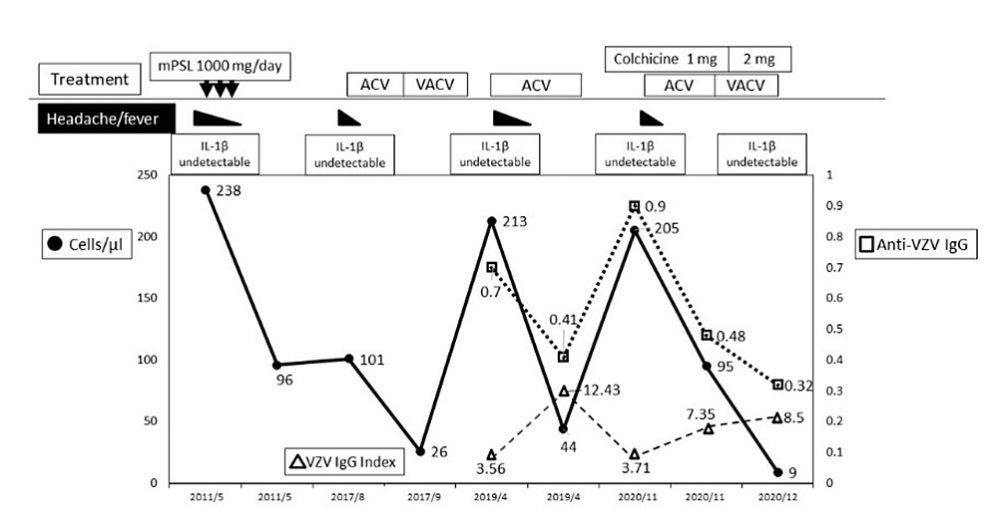
The patient’s clinical course This figure shows the changes in the patient’s clinical course at admission. The number of cells and the titer of anti-VZV IgG (EIA) in CSF repeatedly increase with each recurrence. The patient was treated with steroids, ACV, or VACA. The IL-1β concentration in the CSF did not increase even in the acute phase of meningitis. The VZV IgG index was > 2. IgG: Immunoglobulin-G; EIA: Enzyme immunoassay; VZV: Varicella-zoster virus; CSF: Cerebrospinal fluid; IL-1β: Interleukin-1 beta; mPSL: Methylprednisolone; ACV: Acyclovir; VACV: Valacyclovir.

We performed several additional analyses. Human immunodeficiency virus-1/-2 antigen and antibody screening tests were negative. The HLA haplotypes of the patient were HLA-B7 (07:02:01)/B52 (52:01:01) and HLA-DR15 (15:02:01)/DR1 (01:01:01). Correspondingly, the patient showed mildly decreased serum IgG (690 mg/dL). IL-1β was not detected in any CSF sample (Figure 2). We considered that the patient had a decreased immune response to VZV. The final patient diagnosis was Mollaret meningitis associated with VZV. We decided not to re-treat with colchicine (2 mg/day) and will administer an antiviral agent in the event of meningitis recurrence.

## Discussion

In 1962, Bruyn et al. proposed diagnostic criteria for Mollaret’s meningitis [8], and Galdi subsequently added some points [9]. These diagnostic criteria are still in use today. HSV-2 was considered a pathogenetic microorganism because of numerous reports of recurrent meningitis caused by HSV-2 [10]. The cause has not been well understood, but reports suggest that the host does not possess sufficient specific immunity to HSV-2 [5]. Only clinical symptoms and CSF findings are mentioned in the criteria of Bruyn et al. (1962) and Galdi (1979). Therefore, although the patient tested negative for HSV-2 infection, both sets of diagnostic criteria were met, and Mollaret meningitis was diagnosed.

The gold standard for the diagnosis of VZV meningitis is the detection of VZV DNA in CSF. However, Gilden et al. asserted that antiviral antibodies are not normally found in CSF and that the presence of anti-VZV IgG in the CSF is sufficient to support a diagnosis of CNS VZV infection without the detection of VZV DNA [11]. Martínez-Martin et al. reported that an IgG index > 2.0 indicates intrathecal IgG synthesis [7], and Inukai et al. made a diagnosis using this value [12]. In our case, the results of the polymerase chain reaction for HSV and VZV in CSF were negative; however, a test for anti-VZV IgG (EIA) in CSF was positive in all aseptic meningitis recurrences, and the VZV IgG index exceeded 2. We believe these findings are consistent with VZV infection.

In our case, IL-1β was not detected in CSF during the acute phase of meningitis. Nour et al. demonstrated that VZV induces the formation of the nucleotide-binding oligomerization domain-like receptor family pyrin domain containing 3 (NLRP3) inflammasome and the associated processing of proinflammatory cytokine IL-1 by activated caspase-1 in infected cells [13]. Furthermore, significant increases have been reported in IL-1β concentration in the CSF of patients with VZV vasculopathy [14]. We suspect that our patient has a deficiency in the release of IL-1β in response to VZV infection of the CNS that caused RAM. Hait et al. revealed that antiviral interferon responses were impaired in most patients with recurrent HSV-2 lymphocytic Mollaret meningitis, which was associated with the dysfunction of protective immunity against HSV-2 in the CNS [4]. Although we did not perform confirmatory functional analyses, we suspected a genetic cytokine release deficiency. In addition, HLA-DR1 (01:01:01), which was detected in this patient, is associated with hypogammaglobulinemia [15]. We surmised that decreased immunoglobulin production may have also contributed to the pathogenesis of Mollaret meningitis in this patient.

Initially, the patient was also suspected of having Mollaret’s meningitis due to FMF. The E148Q mutation is believed to be a benign polymorphism and not a disease-causing mutation [16]. Capron et al. proposed the following diagnostic criteria for recurrent meningitis related to FMF: (a) episodes of RAM due to FMF should be accompanied by other clinical or biological features of FMF attacks, (b) colchicine should prevent or lessen episodes, and (c) other classical causes of RAM should be excluded [2]. We considered that the patient did not meet these criteria. The main pathology of FMF is the overproduction of IL-1β. However, Koga et al. reported that serum IL-1β was not elevated in patients with FMF, while other related cytokines were elevated in serum. Koga et al. speculated that this was because IL-1β was produced mainly in inflamed local tissues, and the serum concentration was therefore not large enough to detect significant differences in the assay we utilized [17].

Although cases in which IL-1β was measured in the CSF of FMF patients have not been reported, IL-1β in CSF increased in patients with CNS neuropsychiatric systemic lupus erythematosus and infections of the CNS compared to healthy controls [18], and its concentration was higher in serum. We initially predicted that IL-1β in CSF would increase in Mollaret meningitis associated with FMF to reflect the inflammation of the affected organs. However, no increase was observed. As mentioned above, we interpret this result as the reflection of a decreased immune response to VZV reactivation in the CNS. At this time, we cannot rule out FMF because IL-1β was not elevated in CSF, given the absence of reports measuring IL-1β in CSF in patients with FMF. However, in the future, if further analysis of IL-1β in CSF as a sign and definitive diagnosis of FMF is conducted in patients with meningitis, CSF IL-1β could be a useful marker in the differentiation of meningitis and the evaluation of the disease state.

## Conclusions

The pathology of Mollaret meningitis, including causative microorganisms, has not been fully clarified. We consider that the dysfunction, probably due to genetic factors, of multiple immune pathways against infectious viruses affecting the CNS underlies the pathogenesis of the disease. We observe that VZV can be a pathogenic microorganism of Mollaret meningitis. We hope that the pathology of this disease will be elucidated and that appropriate treatment will be established.
